# Actor or recipient role matters: Neural correlates of self‐serving bias

**DOI:** 10.1002/brb3.2013

**Published:** 2021-01-26

**Authors:** Xiaoyan Wang, Li Zheng, Lin Li, Peng Sun, Xiuyan Guo

**Affiliations:** ^1^ School of Psychology Sichuan Normal University Chengdu China; ^2^ School of Psychology and Cognitive Science East China Normal University Shanghai China; ^3^ Key Laboratory of Brain Functional Genomics Ministry of Education Shanghai Key Laboratory of Brain Functional Genomics East China Normal University Shanghai China; ^4^ National Demonstration Center for Experimental Psychology Education East China Normal University Shanghai China; ^5^ Shanghai Key Laboratory of Magnetic Resonance and School of Psychology and Cognitive Science East China Normal University Shanghai China

**Keywords:** actor/recipient role, dmPFC, self‐serving bias (SSB)

## Abstract

**Introduction:**

When involved in interpersonal events, people often play the role of an initiative actor (e.g., “I hit Tom”) or a passive recipient (e.g., “Paul hit me”). Numerous studies have documented that people manifest a self‐serving bias (SSB), that is, they tend to attribute positive interpersonal events to themselves and negative events to other external factors. Recent studies have identified the neural regions associated with the SSB; yet little is known about the neural mechanism of its modulation by the actor or recipient role.

**Methods:**

In this study, participants were scanned while they attributed the positive or negative events in which the self played the actor or recipient role.

**Results:**

The results showed that people manifested more SSB than non‐SSB (NONSSB) attributions and spent less time on making the former. Importantly, more SSB attributions and shorter reaction times were found in the actor than in the recipient condition. Greater activity in the dorsomedial prefrontal cortex (dmPFC) was observed in responding to NONSSB than SSB attributions only in the actor condition. Furthermore, the greater the difference in dmPFC activity in responding to NONSSB and SSB attributions, the smaller the difference in corresponding attribution response.

**Conclusion:**

The results suggest that people prefer making heuristic SSB attributions, and more cognitive resources are needed when they make NONSSB attributions. The activity of the dmPFC may be associated with inhibiting the heuristic SSB, especially when they play the actor role at interpersonal events.

## Graphical abstract

People manifested the self‐serving bias (SSB) in social situations. The SSB effect was greater in the actor than in the recipient condition. Greater dmPFC activated in NONSSB than SSB attributions in the actor condition. Activity of the dmPFC may be associated with inhibiting the heuristic SSB, especially when we play the actor role at interpersonal events.

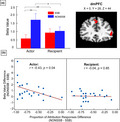

## 1 INTRODUCTION

People tend to make claims that cast the self in a favorable light (Cai et al., 2016; Chavez et al., 2016; Hughes & Beer, 2012; Korn et al., 2012; Sharot et al., 2007). This tendency can be manifested by the self‐serving bias (SSB) in attribution. That is, people are more likely to attribute positive events to themselves but attribute negative events to other factors in social situations (Blackwood et al., 2003; Mezulis et al., 2004). One explanation for the SSB is that people have the self‐enhancement motivation to enhance the positivity or diminish the negativity of their self‐concept (Sedikides et al., 2015; Sedikides & Gregg, 2008). Yet people also have other motives associated with the self‐concept, such as the self‐assessment motivation, which prefers accurate information about the self (Duval & Silvia, 2002). Thus, sometimes, people make non‐SSB (NONSSB) attributions, that is, they make external attributions of positive events and internal attributions of the negative ones (Wang, Zheng, Cheng, et al., 2015; Wang et al., 2017). How people modulate their attributions under various situations is still an open and interesting question.

Previous studies have found that SSB is modulated by the actor or recipient role (Wang, Zheng, Cheng, et al., 2015; Wang et al., 2017). When involved in interpersonal events, people often play the role of an initiative actor (e.g., “I hit Tom”) or a passive recipient (e.g., “Paul hit me”). As an actor, the individual takes the initiative in social interactions when they actively perform the actions in interpersonal events. As a recipient, the individual passively receives uncontrollable actions in interpersonal events. Compared with the passive recipient role, people manifest more SSB when they play the actor role. Researchers have argued that when the self is the grammatical subject of the sentence describing interpersonal events in an actor condition, it has more salience than in the recipient condition. Thus, the actor is more likely to be considered the cause of emotional interpersonal events (Kasof & Lee, 1993), and the actor might be supposed to take more responsibility for the interpersonal events relative to the recipient. In order to enhance the positivity or diminish the negativity of their self‐concept (Sedikides & Alicke, 2012), people might attribute more positive events and less negative events to themselves in the actor condition relative to the recipient condition (Wang, Zheng, Cheng, et al., 2015; Wang et al., 2017). Alternatively, because of the higher salience of the self in the actor condition than in the recipient condition, people might have a higher self‐awareness level. When self‐awareness is heightened, people take more notice of their self‐concept, and the self‐enhancement motivation is increased (Duval & Silvia, 2002). Thus, people might manifest more SSB in the actor condition than in the recipient condition.

Neuroimaging studies have investigated the neural correlates of the SSB by distinguishing individuals' SSB attributions from NONSSB attributions (Blackwood et al., 2003; Seidel et al., 2010, 2012). SSB attributions refer to the combined responses of self‐attributions for positive events and other‐factor attributions for negative events, while NONSSB attributions refer to the combined responses of other‐factor attributions for positive events and self‐attributions for negative events. Previous findings have shown that SSB attributions are associated with activities of the striatum (including the caudate and putamen), dorsal anterior cingulate cortex, and orbitofrontal cortex (OFC). These regions are considered to represent the personal value of information from diverse sources, ranging from social rewards (e.g., positively biased self‐views, positive feedback on personality traits) to nonsocial rewards (e.g., food, money; Blackwood et al., 2003; Seidel et al., 2010). Meanwhile, neural activities in the prefrontal cortex, such as the dorsomedial prefrontal cortex (dmPFC) and ventrolateral prefrontal cortex (vlPFC; Blackwood et al., 2003; Seidel et al., 2012), are associated with NONSSB. These regions may be engaged in cognitive control to suppress the heuristic self‐serving attributions (Blackwood et al., 2003; Seidel et al., 2012). These findings suggest that, although SSB attributions are considered helpful for protecting self‐esteem and maintaining mental health, people also make NONSSB attributions to gain accurate self‐knowledge according to the situation, and the brain regions associated with SSB and NONSSB attributions are different. Given that the SSB or NONSSB effect differs between the actor and the recipient condition (Wang et al., 2017), we hypothesize that these brain regions associated with SSB or NONSSB attributions are modulated by the actor or recipient role.

To examine the above hypothesis, the behavioral and neuroimaging data from Wang, Zheng, Cheng, et al. (2015) were used in the present study. In their study, the role that “self” played (actor or recipient) in an interpersonal event was manipulated. Based on that data, the effect of the actor/recipient role on the self‐serving bias and its underlying neural correlates could be examined in the present study. Their work focused on uncovering the difference between people's behavioral and neural responses to the self and others in interpersonal events. They found that the dmPFC was more activated when evaluating self‐related events relative to other‐related events, and the difference in dmPFC activity in responding to the self and to others was positively correlated with individuals' reaction times in the recipient condition (Wang, Zheng, Cheng, et al., 2015). They also argued that people might be inclined to employ more cognitive resources to rate themselves in the recipient condition compared with the actor condition (Wang, Zheng, Cheng, et al., 2015). These findings revealed that people's patterns of self‐processing are closely correlated with the actor or recipient role they play in interpersonal events. However, they did not illustrate the neural mechanism underlying the effect of the actor or recipient role on the SSB.

In the present study, we aimed to examine how the actor/recipient role affects the behavioral and neural mechanism of the SSB. Based on the previous studies on SSB (Blackwood et al., 2003; Seidel et al., 2010, 2012), we distinguished individuals' SSB from NONSSB attributions for positive and negative interpersonal events in both actor and recipient conditions. For self‐related interpersonal events, individuals' high self‐relevance attributions of positive events or low self‐relevance attributions of negative events were called SSB attributions; the reverse attribution patterns were called NONSSB attributions (Blackwood et al., 2003; Seidel et al., 2010, 2012). At the behavioral level, we expected that, compared with the recipient condition, people would manifest more SSB attributions in the actor condition. At the neural level, brain regions associated with reward would be more activated when people made more SSB attributions, while regions associated with cognitive control would be more activated when people made more NONSSB attributions. These brain activities associated with SSB or NONSSB attributions would be modulated by the actor/recipient role that “self” played in interpersonal events.

## 2 METHODS

### 2.1 Participants

Twenty‐nine right‐handed volunteers were recruited in compliance with the human subject regulations of the Ethical Committee of East China Normal University. All participants provided informed consent before scanning and were paid RMB 50 for their participation. Five participants had to be excluded from the analysis: Four participants were excluded due to excessive head movements and one made no response in one experimental condition. The remaining 24 participants included in the data analysis consisted of 12 females, aged from 20 to 30 years, *M* = 24.0, *SD* = 2.56. All participants reported no abnormal neurological history and had normal or corrected‐to‐normal vision.

### 2.2 Behavioral paradigm

Participants were randomly presented with 160 one‐sentence interpersonal events (80 positive and 80 negative). Half of them were self‐relevant events, where “self” was randomly assigned as an actor (e.g., “I praise Lisa”) or a recipient (e.g., “Paul hit me”). In each trial, the participant was asked to read a sentence and to rate “How likely is it that I am that kind of person?” on a 4‐point scale within 5 s by pressing the corresponding button (1 = very unlikely, 2 = moderately unlikely, 3 = moderately likely, 4 = very likely). Additionally, there were 80 other‐relevant events (e.g., “Mary hits Lisa”). Participants were asked to imagine the event and rate “How likely is it that the actor (e.g., Mary) [or recipient (e.g., Lisa)] is that kind of person?” Because the two persons in each interpersonal event were unfamiliar to the participants, they were asked to answer according to most cases in social situations. Following fMRI scanning, participants completed the Rosenberg Self‐Esteem Scale (Rosenberg, 1989).

### 2.3 fMRI data acquisition

All images were collected on a 3T Siemens scanner at the Functional MRI Lab (East China Normal University, Shanghai). Functional images were acquired using a gradient‐echo echo‐planar imaging (EPI) sequence (TR = 2,200 ms, TE = 30 ms, FOV = 220, matrix size = 64 × 64, slice thickness = 3 mm, gap = 0.3 mm) with each volume consisting of 35 slices. A high‐resolution T1‐weighted image was also acquired from each participant (TR = 1900 ms, TE = 3.42 ms, 192 slices, slice thickness = 1 mm, FOV = 256 mm, matrix size = 256 × 256) before the functional run.

### 2.4 fMRI data preprocessing and statistical analyses

Data analyses were conducted with SPM8 (Wellcome Department of Cognitive Neurology, London). Preprocessing included discarding the first five functional images to allow for scanner equilibrium effects. The remaining 569 functional images were reoriented according to Anterior and Posterior Commissure (AC‐PC) plane, spatially realigned to the first volume, spatial normalization into the Montreal Neurological Institute (MNI) template (resampled at 2 × 2 × 2 mm^3^ voxels), and spatial smoothing (using an 8‐mm full‐width half maximum isotropic Gaussian kernel). A high‐pass filter with a cutoff period of 128 s was applied.

At the first level, to calculate the SSB attributions and NONSSB attributions of a participant in actor and recipient conditions, four conditions were defined according to Role (Actor vs. Recipient) and AttriBias (SSB vs. NONSSB). SSB attributions referred to the combined responses of high self‐relevance attribution of positive events (3 or 4 response) and low self‐relevance attribution of negative events (1 or 2 response) by the participant. NONSSB attributions referred to the combined effects of low self‐relevance attribution of positive events (1 or 2 response) and high self‐relevance attribution of negative events (3 or 4 response) by the participant (Blackwood et al., 2003; Seidel et al., 2012). These four conditions were modeled using a canonical hemodynamic response function with a temporal derivative. We chose the onset of the stimulus as the onset time point and the reaction time (RT) from the stimulus onset to button press as the duration (epoch with variable time length). One regressor modeling the other‐relevant interpersonal events, six modeling movement‐related variance, and one modeling the overall mean were also employed in the design matrix. A general linear model analysis created four contrast images for each participant summarizing differences of interest. The four first‐level contrast images from each participant were then analyzed at the second level employing a random‐effects model (flexible factorial design in SPM8). We used *t*‐contrasts to analyze the effects of Role, AttriBias, and the interaction between them.

Parametric analyses were also conducted to assess how brain activities were modulated by the level of self‐serving bias attributions in positive and negative events separately, using attribution ratings as the parametric modulators. The resulting participant‐specific estimates of the parametric regressor at each voxel were then entered into a second‐level one sample *t* test treating participants as a random variable. Regions showing increased activations in response to self‐related positive events with the increment of attribution ratings and to negative events with the reduction of attribution ratings were identified separately to evaluate the brain activities associated with the self‐serving bias.

Areas of activation were identified as significant only if they met the thresholded of *p* < .05, FWE‐corrected for multiple comparisons at the cluster level with an underlying voxel level of *p* < .001 (uncorrected), unless otherwise indicated. The MarsBaR toolbox was used to extract beta‐values from the activated brain regions (Brett et al., 2002).

## 3 RESULTS

### 3.1 Behavioral data

Each attribution rating was subdivided into a low self‐relevance (events that received a 1 or 2 response from the participant) or high self‐relevance (events that received a 3 or 4 response from the participant) condition. The proportion of attributions for each condition made by the 24 participants was analyzed to examine the self‐serving bias. Then, the proportions of each participant's SSB (high self‐relevance attributions of positive events and low self‐relevance attributions of negative events) and NONSSB (low self‐relevance attributions of positive events and high self‐relevance attributions of negative events) attributions in actor and recipient conditions were calculated and compared. Additionally, the corresponding reaction time (RT) for each condition made by the 24 participants was also calculated and analyzed.

#### 3.1.1 Attribution rating

In both actor and recipient conditions, paired‐sample *t* tests revealed that participants made more high self‐relevance responses relative to low self‐relevance responses to positive events (actor: *t*(23) = 9.29, *p* < .001; recipient: *t*(23) = 8.19, *p* < .001), and this pattern was reversed in responding to negative events (actor: *t*(23) = 15.96, *p* < .001; recipient: *t*(23) = 10.37, *p* < .001). These results indicated that people manifested the SSB in both actor and recipient conditions.

Furthermore, paired‐sample *t* tests revealed that participants' SSB attributions were significantly greater than NONSSB attributions in both the actor (*t*(23) = 13.15, *p* < .001) and recipient (*t*(23) = 13.31, *p* < .001) conditions. However, the differences between the proportions of SSB and NONSSB attributions were significantly greater in the actor relative to the recipient condition (*t*(23) = 3.22, *p* = .004). In addition, we found that participants made more SSB attributions and fewer NONSSB attributions in the actor condition relative to the recipient condition (SSB attributions: *t*(23) = 3.22, *p* = .004; NONSSB attributions: *t*(23) = 3.21, *p* = .004).

Notably, correlation analysis was conducted to explore the relationship between self‐esteem and the SSB attributions. A positive correlation was observed between people's level of self‐esteem and the proportion of SSB attributions in the actor condition, *r* = .42, *p* = .04, 95% CI = (0.001, 0.047); however, there was no significant correlation in the recipient condition, *r* = .31, *p* = .14, 95% CI = (−0.007, 0.050).

#### 3.1.2 Reaction time

For the RT (see Table 1), a 2 (Role: actor vs. recipient) × 2 (Valence: positive vs. negative) × 2 (Self‐relevance: high vs. low) repeated measure ANOVA revealed main effects of Role (*F*(1, 23) = 12.71, *p* = .002, $\eta_{p}^{2}=0.36$) and Valence (*F*(1, 23) = 6.41, *p* = .02, $\eta_{p}^{2}=0.22$). Additionally, there was a significant interaction between valence and self‐relevance (*F*(1, 23) = 17.39, *p* < .001, $\eta_{p}^{2}=0.43$). No other main effects or interactions were significant, all *F* < 1.01, all *p* > .33. Further simple effect analysis revealed that participants' high self‐relevance responses were faster than their low self‐relevance responses for positive events (*t*(23) = 3.82, *p* = .001), while their high self‐relevance responses were slower than their low self‐relevance responses for negative events (*t*(23) = 2.78, *p* = .01).

**TABLE 1:** Means and standard deviations of participants' RT (ms) across conditions

	Positive		Negative	
	HSR	LSR	HSR	LSR
Actor	2,183.56 ± 507.29	2,406.65 ± 490.58	2,496.35 ± 681.14	2,216.39 ± 429.36
Recipient	2,254.57 ± 552.54	2,632.72 ± 628.79	2,705.77 ± 752.96	2,484.15 ± 489.31

Abbreviations: HSR, high self‐relevance; LSR, low self‐relevance.

Furthermore, we calculated each participant's SSB and NONSSB attribution reaction time in the actor and recipient conditions. A 2 (Role: actor vs. recipient) × 2 (AttriBias: SSB vs. NONSSB) repeated measure ANOVA revealed significant main effects of Role (*F*(1, 23) = 10.25, *p* = .004, $\eta_{p}^{2}=0.31$) and AttriBias (*F*(1, 23) = 13.43, *p* = .001, $\eta_{p}^{2}=0.37$). There was no significant interaction between Role and AttriBias (*F*(1, 23) = 0.10, *p* = .76). Pairwise comparisons revealed that people made faster responses in the actor (*M* = 2,326.74 ms) relative to the recipient condition (*M* = 2,519.30 ms; *p* = .004), and they made faster responses for SSB (*M* = 2,285.67 ms) than for NONSSB attributions (*M* = 2,560.07 ms; *p* = .001).

### 3.2 fMRI data

#### 3.2.1 Main effects and interaction

For the main effects, the SSB versus NONSSB contrast revealed the precentral gyrus (MNI: −22 −18 60). The NONSSB versus SSB contrast revealed the dorsomedial prefrontal context (dmPFC; MNI: 2 36 50, −4 42 44) and inferior frontal gyrus (MNI: −54 22 4). The Actor versus Recipient contrast revealed the fusiform gyrus (MNI: 24 −68 −4) and inferior parietal gyrus (MNI: −44 −26 44). The reverse contrast revealed the angular gyrus (MNI: −44 −52 24).

The interaction between AttriBias and Role was computed by the (Recipient [SSB‐NONSSB]‐Actor [SSB‐NONSSB]) contrast and the reverse contrast. The results showed that the middle temporal gyrus (MNI: 68 −34 2), supplementary motor area (MNI: 6 24 56), dmPFC (MNI: 0 28 44), inferior frontal gyrus (MNI: 52 34 2), postcentral gyrus (MNI: 34 −28 60), and middle cingulate cortex (MNI: −2 −32 46) were activated in (Recipient [SSB‐NONSSB]‐Actor [SSB‐NONSSB]) contrast. No regions survived the reverse contrast (Table 2).

**TABLE 2:** Identification of BOLD signal changes association with main effects of AttriBias, Role, and the AttriBias × Role interaction

Brain region		MNI						*T* value	Voxels
		X		Y		Z			
SSB‐NONSSB									
L	Precentral gyrus	−22		−18		60		4.31	235
NONSSB‐SSB									
R	Dorsomedial prefrontal context	2		36		50		5.67	1883
		−*4*		*42*		*44*		*5.27*	
L	Inferior frontal gyrus	−54		22		4		4.58	351
Actor‐Recipient									
R	Fusiform gyrus	24		−68		−4		5.20	255
L	Inferior parietal gyrus	−44		−26		44		5.09	1,840
Recipient‐Actor									
L	Angular gyrus	−44		−52		24		4.51	244
Actor (SSB‐NONSSB)–Recipient (SSB‐NONSSB)									
None									
Recipient (SSB‐NONSSB)–Actor (SSB‐NONSSB)									
R	Middle temporal gyrus		68		−34		2	4.41	396
R	Supplementary motor area		6		24		56	4.14	245
	Dorsomedial prefrontal context		*0*		*28*		*44*	*3.95*	
R	Inferior frontal gyrus		52		34		2	4.13	303
R	Postcentral gyrus		34		−28		60	3.99	277
L	Middle cingulate cortex		−2		−32		46	3.76	300

Note

All reported clusters are cluster‐level family wise error (FWE) corrected for multiple comparisons at *p* < .05 with an underlying voxel level of *p* < .001 (uncorrected).

Abbreviations: L, left hemisphere; NONSSB, non‐self‐serving bias; R, right hemisphere; SSB, self‐serving bias.

**FIGURE 1:**
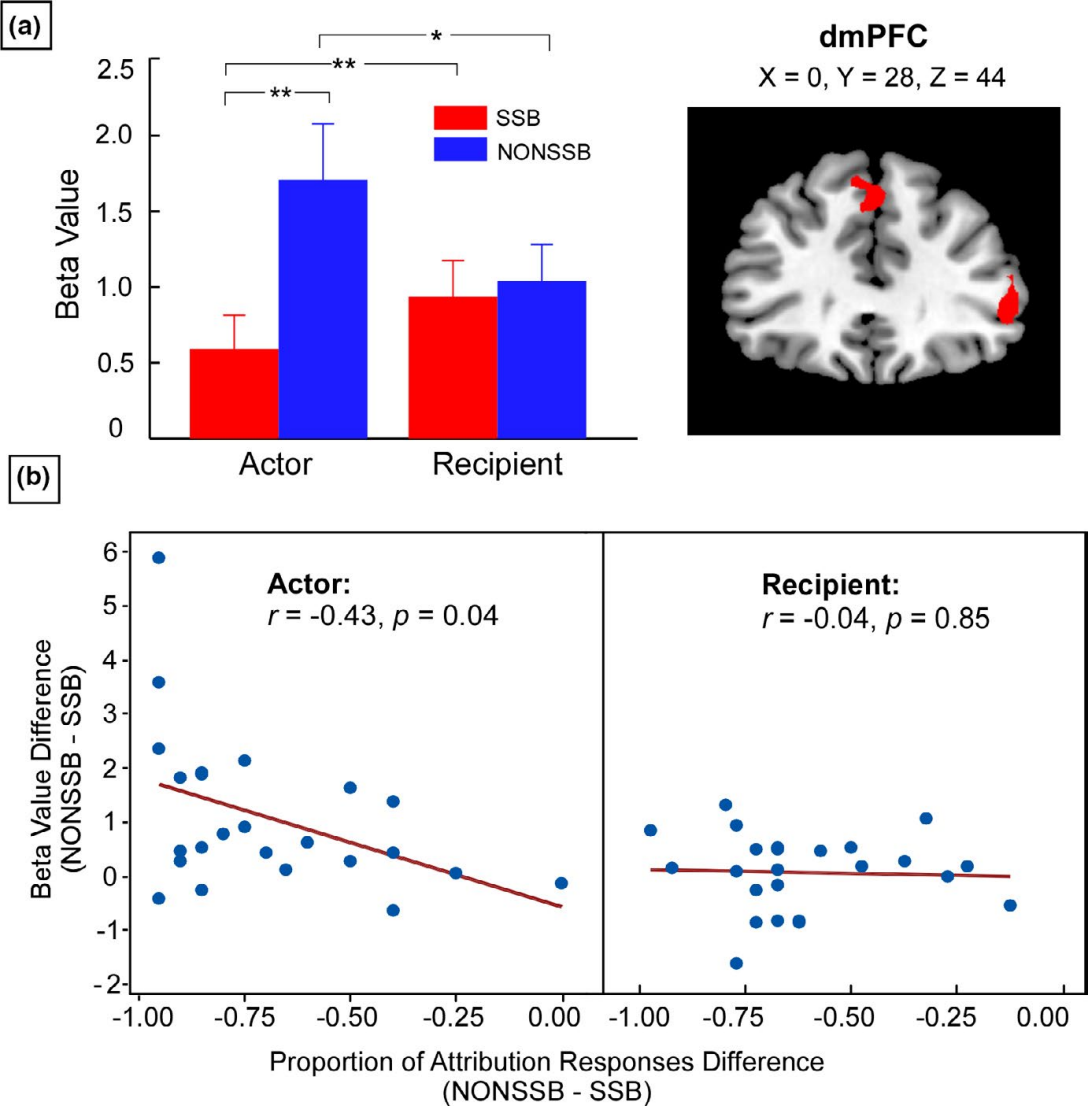
Parameter estimates of the dorsomedial prefrontal cortex (dmPFC). (a) Brain activity of the dmPFC (MNI: 0 28 44) was greater for NONSSB attributions than for SSB attribution in both the actor and the recipient conditions. In addition, the dmPFC activation was greater for NONSSB attributions in the actor condition than in the recipient condition, while it was greater for SSB attributions in the recipient condition than in the actor condition (**p < .01, *p < .05). Error bars indicated standard errors of mean beta‐values. (b) The beta value difference (NONSSB–SSB) in dmPFC activity was negatively correlated with the corresponding attribution difference across all participants in the actor rather than in the recipient condition. NONSSB, non‐self‐serving bias; SSB, self‐serving bias

The cluster located in the dmPFC overlapped between the contrast of (NONSSB‐SSB) and the contrast of (Recipient [SSB‐NONSSB]‐Actor [SSB‐NONSSB]). Parameter estimates across the dmPFC (MNI: 0 28 44) were extracted. A 2 (Role: actor vs. recipient) × 2 (AttriBias: SSB vs. NONSSB) repeated measures ANOVA revealed a significant interaction between Role and AttriBias (*F*(1, 23) = 11.32, *p* = .003, $\eta_{p}^{2}=0.33$). Furthermore, simple effect analysis revealed that activity in the dmPFC was greater for NONSSB attributions than for SSB attributions in the actor condition (*t*(23) = 3.79, *p* = .001) but not in the recipient condition (*t*(23) = 0.70, *p* = .49; Figure 1a). Additionally, we also found that activity in the dmPFC was greater for NONSSB attributions in the actor condition compared with the recipient condition (*t*(23) = 2.41, *p* = .02), while activity in dmPFC was greater for SSB attributions in the recipient condition compared with the actor condition (*t*(23) = 4.00, *p* = .001).

Correlation analysis showed a significant negative correlation between the beta value differences (NONSSB‐SSB) of dmPFC (MNI: 0 28 44) activity and the attribution proportion differences (NONSSB‐SSB) in the actor condition, *r* = −.43, *p* = .04, 95% CI = (−0.116, −0.004); however, the correlation was not significant in the recipient condition, *r* = −.04, *p* = .85, 95% CI = (−0.04, 0.033; Figure 1b).

#### 3.2.2 Parametric analyses of attribution rating

Parametric analyses revealed that, for self‐relevant positive events, the insula (MNI: −38 −2 4) showed increased activation when participants reported higher self‐relevance. The left dorsolateral prefrontal cortex (dlPFC; MNI: −52 28 34) showed decreased activation when participants reported higher self‐relevance. Meanwhile, for self‐relevant negative events, the putamen (MNI: 30 −6 0) showed increased activation when participants reported lower self‐relevance (Table 3).

**TABLE 3:** Regions showing increased and decreased activations for self‐relevant positive and negative interpersonal events with higher attribution ratings

	Brain region	MNI			*t* value	Voxels
		X	Y	Z		
Self‐relevant positive events						
Increased						
L	Precentral gyrus	−32	−26	66	8.85	3,896
L	Insula	−38	−2	4	6.40	213
Decreased						
L	Lingual gyrus	−8	−66	−8	8.75	1577
R	Precentral gyrus	34	−16	64	8.16	5,346
L	Supplementary motor area	−2	10	58	5.46	630
L	Left dorsolateral prefrontal cortex	−52	28	34	4.56	206
Self‐relevant negative events						
Increased						
L	Postcentral gyrus	−36	−24	46	6.52	979
Decreased						
R	Postcentral gyrus	44	−28	60	9.95	4,268
	*Putamen*	*30*	−*6*	*0*	*9.42*	

Note

All reported clusters are cluster‐level family wise error (FWE) corrected for multiple comparisons at *p* < .05 with an underlying voxel level of *p* < .001 (uncorrected).

Abbreviations: L, left hemisphere; R, right hemisphere.

## 4 DISCUSSION

The present study aimed to explore how the actor or recipient role modulates people's SSB and the underlying neural mechanisms. Behavioral results confirmed that people manifested the SSB in attribution. Importantly, people showed more SSB and shorter reaction time in the actor relative to the recipient condition, and the proportion of SSB attributions was positively correlated with self‐esteem in the actor condition but not in the recipient condition. At the neural level, the activity of the dmPFC was greater while responding to NONSSB than SSB attributions in the actor condition but not in the recipient condition. Moreover, only in the actor condition, we found the greater the difference in dmPFC activity in responding to NONSSB attributions and SSB attributions, the smaller the difference in the corresponding attribution response. Additionally, more SSB was found to be associated with higher activities in the putamen and insula, and less SSB was associated with higher activity in the dlPFC.

Behavioral results revealed that people manifested the SSB in attribution, which was consistent with previous work showing that the SSB is pervasive in the general population (Blackwood et al., 2003; Mezulis et al., 2004). Importantly, people manifested more SSB in the actor condition relative to the recipient condition. Compared with passive recipients, people who took the initiative to perform the interpersonal actions were more likely to be considered as the cause of the interpersonal events (Kasof & Lee, 1993). In order to maintain their self‐esteem, people who played the actor role had more desire to attribute more positive events and fewer negative events to themselves compared with the passive recipient condition. In addition, we observed a shorter reaction time in the actor condition relative to the recipient condition. Our findings further suggested that, when people took the initiative actor role, their attribution responses might be more heuristic rather than deliberative. In that situation, the desire to maintain positive self‐esteem might be greater than other motivations, such as accurate self‐evaluation. Additionally, we found that the SSB is positively correlated with the level of self‐esteem in the actor condition rather than in the recipient condition. It is worth noting that although the correlation was not statistically significant in the recipient condition, the confidence intervals overlapped between the actor and recipient conditions. This suggested that whether the actor or recipient role is played, people with a higher level of self‐esteem might manifest more SSB attributions. The positive relationship between self‐esteem and the self‐serving cognition has been well‐documented in previous studies where people made self‐reference judgments or responsibility attributions (Somerville et al., 2010; Yang et al., 2014). Future studies could further confirm the difference between the actor and the recipient role.

At the neural level, we found greater dmPFC activity for NONSSB than SSB attributions in the actor condition. Activity of the dmPFC has been associated with self‐evaluation and self‐related reappraisal (D'Argembeau et al., 2007; Han et al., 2010; Korn et al., 2012; Lemogne et al., 2011; Northoff & Bermpohl, 2004). In addition, it is involved in conflict monitoring and cognitive control (D'Argembeau et al., 2012; Han et al., 2010; Seidel et al., 2012). Given that individuals need employ more cognitive resources to make the deliberative NONSSB attributions compared with the heuristic SSB attributions (Blackwood et al., 2003; Seidel et al., 2012), our results suggested that the activity of the dmPFC might be associated with inhibiting the SSB. Further evidence came from the negative correlation between dmPFC activation difference (NONSSB‐SSB) and the corresponding attribution response difference in the actor condition. It is possible that people prefer to make heuristic SSB attributions in the actor condition. Otherwise, making NONSSB attributions conflicts with their self‐enhancement motivation and require additional cognitive resources. Thus, greater activity of the dmPFC may be involved in reducing the heuristic SSB attributions in the actor condition.

Parametric analyses revealed that greater putamen activity in response to negative events was associated with less self‐relevance. Putamen activity has repeatedly been found to be associated with stimuli/actions that have a rewarding value (Blackwood et al., 2003; Cascio et al., 2015; Dutcher et al., 2016; Liu et al., 2016; Seidel et al., 2010). The putamen activity in the present study may have been involved in tracking the rewarding values of SSB attributions by isolating negative events from the self. Additionally, we found that the insula was positively related to the level of endorsing positive events as self‐relevant, and the dlPFC was negatively related to the level of endorsing positive events as self‐relevant. The insula has been proposed to be involved in self‐related emotion processing (Deen et al., 2011) and the interaction between emotional arousal and valence (Citron et al., 2014; Wang, Zheng, Li, et al., 2015). This suggests that self‐related positive events, which are in line with people's expectations, are likely to attract the attention of individuals. Meanwhile, the dlPFC has been implicated in control‐related processes (Guo et al., 2013; Sanfey et al., 2003). It may have been associated with inhibiting the heuristic SSB in the present study. Taken together with prior results, our present findings suggest that the neural mechanism of the SSB may differ between positive and negative interpersonal events. Given that the SSB is so great in human cognition (Mezulis et al., 2004) and people make external attributions for most negative events, the NONSSB attributions of positive or negative events are so low that the statistical power may have been reduced in the present fMRI design. Therefore, based on previous studies (Blackwood et al., 2003; Seidel et al., 2010, 2012), we combined the internal attribution of positive events and external attribution of negative events to explore the neural mechanism of the SSB. Future studies could further explore the SSB difference between positive and negative events by using more events or other experimental paradigms.

In conclusion, our results confirm that the actor or recipient role affects a person's SSB. People manifested more SSB and a shorter reaction time in the actor relative to the recipient condition. Importantly, only when the self played the role of an actor, more dmPFC engagement was observed in NONSSB relative to SSB attributions. Furthermore, the difference in dmPFC activity in responding to NONSSB attributions and SSB attributions was negatively correlated with the corresponding difference in attribution response. These results provide evidence that in an actor condition, people may prefer to make heuristic SSB attributions, and more cognitive resources are needed when they make NONSSB attributions. The activity of the dmPFC may be associated with inhibiting the SSB, especially when the self plays the role of an actor in interpersonal events.

## CONFLICT OF INTEREST

None declared.

## AUTHOR CONTRIBUTION

Xiaoyan Wang, Li Zheng and Xiuyan Guo devised the concept and supervised the study. Xiaoyan Wang collected the data. Xiaoyan Wang, Li Zheng, Lin Li, Peng Sun and Xiuyan Guo joined in the interpretation of data. Xiaoyan Wang and Li Zheng carried out the writing of the manuscript.

### Peer Review

The peer review history for this article is available at https://publons.com/publon/10.1002/brb3.2013.

## Data Availability

The data that support the findings of this study are available from the corresponding author upon reasonable request.

## References

[brb32013-bib-0001] Blackwood, N. J. , Bentall, R. P. , ffytche, D. H. , Simmons, A. , Murray, R. M. , & Howard, R. J. (2003). Self‐responsibility and the self‐serving bias: An fMRI investigation of causal attributions. NeuroImage, 20(2), 1076–1085. 10.1016/S1053-8119(03)00331-8 14568477

[brb32013-bib-0002] Brett, M. , Anton, J.‐L. , Valabregue, R. , & Poline, J.‐B. (2002). Region of interest analysis using an SPM toolbox. Paper presented at the 8th International Conference on Functional Mapping of the Human Brain, Sendai, Japan.

[brb32013-bib-0003] Cai, H. , Wu, L. , Shi, Y. , Gu, R. , & Sedikides, C. (2016). Self‐enhancement among Westerners and Easterners: A cultural neuroscience approach. Social Cognitive and Affective Neuroscience, 11(10), 1569–1578. 10.1093/scan/nsw072 27217110 PMC5040913

[brb32013-bib-0004] Cascio, C. N. , O'Donnell, M. B. , Tinney, F. J. , Lieberman, M. D. , Taylor, S. E. , Strecher, V. J. , & Falk, E. B. (2015). Self‐affirmation activates brain systems associated with self‐related processing and reward and is reinforced by future orientation. Social Cognitive and Affective Neuroscience, 11(4), 621–629. 10.1093/scan/nsv136 26541373 PMC4814782

[brb32013-bib-0005] Chavez, R. S. , Heatherton, T. F. , & Wagner, D. D. (2016). Neural population decoding reveals the intrinsic positivity of the self. Cerebral Cortex, 27(11), 5222–5229. 10.1093/cercor/bhw302 PMC6075222 27664966

[brb32013-bib-0006] Citron, F. M. M. , Gray, M. A. , Critchley, H. D. , Weekes, B. S. , & Ferstl, E. C. (2014). Emotional valence and arousal affect reading in an interactive way: Neuroimaging evidence for an approach‐withdrawal framework. Neuropsychologia, 56, 79–89. 10.1016/j.neuropsychologia.2014.01.002 24440410 PMC4098114

[brb32013-bib-0007] D'Argembeau, A. , Jedidi, H. , Balteau, E. , Bahri, M. , Phillips, C. , & Salmon, E. (2012). Valuing one's self: Medial prefrontal involvement in epistemic and emotive investments in self‐views. Cerebral Cortex, 22(3), 659–667. 10.1093/cercor/bhr144 21680845

[brb32013-bib-0008] D'Argembeau, A. , Ruby, P. , Collette, F. , Degueldre, C. , Balteau, E. , Luxen, A. , Maquet, P. , & Salmon, E. (2007). Distinct regions of the medial prefrontal cortex are associated with self‐referential processing and perspective taking. Journal of Cognitive Neuroscience, 19(6), 935–944. 10.1162/jocn.2007.19.6.935 17536964

[brb32013-bib-0009] Deen, B. , Pitskel, N. B. , & Pelphrey, K. A. (2011). Three systems of insular functional connectivity identified with cluster analysis. Cerebral Cortex, 21(7), 1498–1506. 10.1093/cercor/bhq186 21097516 PMC3116731

[brb32013-bib-0010] Dutcher, J. M. , Creswell, J. D. , Pacilio, L. E. , Harris, P. R. , Klein, W. M. P. , Levine, J. M. , Bower, J. E. , Muscatell, K. A. , & Eisenberger, N. I. (2016). Self‐affirmation activates the ventral striatum. Psychological Science, 27(4), 455–466. 10.1177/0956797615625989 26917214

[brb32013-bib-0011] Duval, T. S. , & Silvia, P. J. (2002). Self‐awareness, probability of improvement, and the self‐serving bias. Journal of Personality and Social Psychology, 82(1), 49–61. 10.1037/0022-3514.82.1.49 11811633

[brb32013-bib-0012] Guo, X. , Zheng, L. , Zhu, L. , Li, J. , Wang, Q. , Dienes, Z. , & Yang, Z. (2013). Increased neural responses to unfairness in a loss context. NeuroImage, 77, 246–253. 10.1016/j.neuroimage.2013.03.048 23562770

[brb32013-bib-0013] Han, S. , Gu, X. , Mao, L. , Ge, J. , Wang, G. , & Ma, Y. (2010). Neural substrates of self‐referential processing in Chinese Buddhists. Social Cognitive and Affective Neuroscience, 5(2–3), 332–339. 10.1093/scan/nsp027 19620181 PMC2894681

[brb32013-bib-0014] Hughes, B. L. , & Beer, J. S. (2012). Medial orbitofrontal cortex is associated with shifting decision thresholds in self‐serving cognition. NeuroImage, 61(4), 889–898. 10.1016/j.neuroimage.2012.03.011 22440647

[brb32013-bib-0015] Kasof, J. , & Lee, J. Y. (1993). Implicit causality as implicit salience. Journal of Personality and Social Psychology, 65(5), 877–891. 10.1037/0022-3514.65.5.877

[brb32013-bib-0016] Korn, C. W. , Prehn, K. , Park, S. Q. , Walter, H. , & Heekeren, H. R. (2012). Positively biased processing of self‐relevant social feedback. Journal of Neuroscience, 32(47), 16832–16844. 10.1523/JNEUROSCI.3016-12.2012 23175836 PMC6621762

[brb32013-bib-0017] Lemogne, C. , Gorwood, P. , Bergouignan, L. , Pélissolo, A. , Lehéricy, S. , & Fossati, P. (2011). Negative affectivity, self‐referential processing and the cortical midline structures. Social Cognitive and Affective Neuroscience, 6(4), 426–433. 10.1093/scan/nsq049 20519253 PMC3150850

[brb32013-bib-0018] Liu, Z. , Li, L. , Zheng, L. , Hu, Z. , Roberts, I. D. , Guo, X. , & Yang, G. (2016). The neural basis of regret and relief during a sequential risk‐taking task. Neuroscience, 327, 136–145. 10.1016/j.neuroscience.2016.04.018 27102420

[brb32013-bib-0019] Mezulis, A. H. , Abramson, L. Y. , Hyde, J. S. , & Hankin, B. L. (2004). Is there a universal positivity bias in attributions? A meta‐analytic review of individual, developmental, and cultural differences in the self‐serving attributional bias. Psychological Bulletin, 130(5), 711–747. 10.1037/0033-2909.130.5.711 15367078

[brb32013-bib-0020] Northoff, G. , & Bermpohl, F. (2004). Cortical midline structures and the self. Trends in Cognitive Sciences, 8(3), 102–107. 10.1016/j.tics.2004.01.004 15301749

[brb32013-bib-0021] Rosenberg, M. (1989). Society and the adolescent self‐image, revised edition. Wesleyan University Press.

[brb32013-bib-0022] Sanfey, A. G. , Rilling, J. K. , Aronson, J. A. , Nystrom, L. E. , & Cohen, J. D. (2003). The neural basis of economic decision‐making in the ultimatum game. Science, 300(5626), 1755–1758. 10.1126/science.1082976 12805551

[brb32013-bib-0023] Sedikides, C. , & Alicke, M. D. (2012). Self‐enhancement and self‐protection motives. In R. M. Ryan , & R. M. Ryan (Eds.), The Oxford handbook of human motivation (pp. 303–322). Oxford University Press.

[brb32013-bib-0024] Sedikides, C. , Gaertner, L. , & Cai, H. (2015). On the panculturality of self‐enhancement and self‐protection motivation: The case for the universality of self‐esteem. In J. E. Andrew (Ed.), Advances in motivation science (Vol. 2, pp. 185–241). Elsevier.

[brb32013-bib-0025] Sedikides, C. , & Gregg, A. P. (2008). Self‐enhancement food for thought. Perspectives on Psychological Science, 3(2), 102–116. 10.1111/j.1745-6916.2008.00068.x 26158877

[brb32013-bib-0026] Seidel, E.‐M. , Eickhoff, S. B. , Kellermann, T. , Schneider, F. , Gur, R. C. , Habel, U. , & Derntl, B. (2010). Who is to blame? Neural correlates of causal attribution in social situations. Social Neuroscience, 5(4), 335–350. 10.1080/17470911003615997 20162490 PMC8019091

[brb32013-bib-0027] Seidel, E.‐M. , Satterthwaite, T. D. , Eickhoff, S. B. , Schneider, F. , Gur, R. C. , Wolf, D. H. , Habel, U. , & Derntl, B. (2012). Neural correlates of depressive realism—An fMRI study on causal attribution in depression. Journal of Affective Disorders, 138(3), 268–276. 10.1016/j.jad.2012.01.041 22377511 PMC3565123

[brb32013-bib-0028] Sharot, T. , Riccardi, A. M. , Raio, C. M. , & Phelps, E. A. (2007). Neural mechanisms mediating optimism bias. Nature, 450(7166), 102–105. 17960136 10.1038/nature06280

[brb32013-bib-0029] Somerville, L. H. , Kelley, W. M. , & Heatherton, T. F. (2010). Self‐esteem modulates medial prefrontal cortical responses to evaluative social feedback. Cerebral Cortex, 20(12), 3005–3013. 10.1093/cercor/bhq049 20351022 PMC2978246

[brb32013-bib-0030] Wang, Q. , Zheng, L. I. , Li, L. , Xu, X. , Cheng, X. , Ning, R. , Dienes, Z. , & Guo, X. (2015). Incidental self‐processing modulates the interaction of emotional valence and arousal. Experimental Brain Research, 233(1), 229–235. 10.1007/s00221-014-4106-7 25262587

[brb32013-bib-0031] Wang, X. , Zheng, L. , Cheng, X. , Li, L. , Sun, L. , Wang, Q. , & Guo, X. (2015). Actor‐recipient role affects neural responses to self in emotional situations. Frontiers in Behavioral Neuroscience, 9, 83. 10.3389/fnbeh.2015.00083 25926781 PMC4397920

[brb32013-bib-0032] Wang, X. , Zheng, L. , Li, L. , Zheng, Y. J. , Sun, P. , Zhou, F. Z. A. , & Guo, X. Y. (2017). Immune to situation: The self‐serving bias in unambiguous contexts. Frontiers in Psychology, 8, 822. 10.3389/fpsyg.2017.00822 28588532 PMC5439270

[brb32013-bib-0033] Yang, J. , Dedovic, K. , Guan, L. , Chen, Y. , & Qi, M. (2014). Self‐esteem modulates dorsal medial prefrontal cortical response to self‐positivity bias in implicit self‐relevant processing. Social Cognitive and Affective Neuroscience, 9(11), 1814–1818. 10.1093/scan/nst181 24396003 PMC4221225

